# Who Should Be the Cardiogeriatrician? A Competency-Based Perspective for an Ageing Cardiovascular Population

**DOI:** 10.3390/jcm15041406

**Published:** 2026-02-11

**Authors:** Rémi Esser, Marine Larbaneix, Alejandro Mondragon, Marlène Esteban, Christine Farges, Vincenzo Palermo, Sophie Nisse Durgeat, Marc Harboun, Olivier Maurou

**Affiliations:** 1 Cardiogeriatrics Department, Hôpital La Porte Verte, 78000 Versailles, France; 2 Cardiology Department, Hôpital Marie Lannelongue, 92350 Le Plessis Robinson, France; v.palermo@ghpsj.fr; 3 NP Medical, Medical Affairs, 33000 Bordeaux, France

**Keywords:** cardiogeriatrics, frailty, older adults, competency-based care, heart failure, shared decision-making

## Abstract

**Background:** Population ageing is reshaping cardiovascular medicine, with older adults increasingly presenting with heart failure, atrial fibrillation, and structural heart disease. Their management is frequently complicated by frailty, multimorbidity, functional impairment, and competing priorities, challenging traditional disease-centred cardiovascular models. **Objective:** To address who should act as the cardiogeriatrician using a competency-based perspective focused on the clinical skills and decision-making capacities required to care for older frail adults. **Methods:** Narrative review of PubMed/MEDLINE literature integrating cardiology, geriatrics, and health services research, including guidelines, consensus statements, observational studies, and conceptual frameworks. **Results:** Cardiogeriatric care models vary widely and are often defined by professional roles rather than competencies. A competency-based framework better captures core requirements, including frailty-informed interpretation, proportionality of care, trajectory-based assessment, and goal-concordant decision-making. Cardiogeriatric expertise may be embodied by different professional profiles. One workable profile is a geriatrician with sustained cardiovascular practice and clearly defined competencies. Cardiology expertise remains essential for diagnostic confirmation, complex decision-making, and interventional care. **Conclusions:** A competency-based conceptualisation of cardiogeriatrics supports collaborative, proportionate, and patient-centred cardiovascular care in ageing populations, without redefining professional boundaries.

## 1. Introduction: Why the Question Matters Now

Population ageing is profoundly reshaping cardiovascular medicine. Very old adults now represent a rapidly expanding proportion of patients with heart failure, atrial fibrillation, and structural heart disease, yet they remain markedly under-represented in cardiovascular trials and guideline-generating evidence [1,2,3]. In this population, clinical outcomes are driven not only by cardiovascular disease severity, but also by frailty, multimorbidity, functional reserve, and cognitive vulnerability, challenging traditional disease-centred care models [4,5,6].

Frailty and functional impairment are now recognised as major, independent determinants of prognosis in older patients with cardiovascular disease, often outweighing classical cardiovascular risk markers [4,5]. These features require integrated approaches that transcend single-organ paradigms and incorporate proportionality of care, treatment tolerance, and patient-centred outcomes into routine decision-making [6,7].

In response to this mismatch, cardiogeriatrics has emerged at the interface of cardiology and geriatrics, aiming to align cardiovascular expertise with geriatric principles. Recent position papers and consensus statements have emphasised the need to integrate frailty assessment, functional evaluation, and shared decision-making into cardiovascular care for older adults [6,7,8,9]. This approach is also supported by the recent consensus document from the European Society of Hypertension (ESH) Working Group on Hypertension and the Heart on hypertensive heart disease in older patients, which recommends periodic assessment of frailty level and functional status to guide treatment decisions in this population [10]. More recently, a competency-based perspective has been proposed to define the skills required of clinicians delivering cardiogeriatric care, highlighting interpretative and decisional abilities that extend beyond procedural or pharmacological expertise alone [11].

In this manuscript, we use “cardiogeriatrics” and “geriatric cardiology” to refer to an integrated clinical approach at the interface of cardiology and geriatrics; terminology may vary across countries and healthcare traditions. We use “cardiogeriatric expertise” in a competency-based sense, i.e., the set of skills required to manage complex cardiovascular disease in older adults (frailty, multimorbidity, functional and cognitive vulnerability), irrespective of professional background.

Despite this growing recognition, cardiogeriatrics remains poorly defined as a clinical discipline. Across healthcare systems, its implementation varies widely, ranging from cardiology-led models with limited geriatric input to geriatric services managing cardiovascular disease without structured cardiology integration. Consequently, the question of who should be the cardiogeriatrician—a cardiologist trained in geriatrics, a geriatrician trained in cardiology, or a hybrid professional—remains largely implicit and unresolved.

This ambiguity is not merely semantic. It has direct implications for clinical decision-making, training pathways, workforce organisation, and the scalability of cardiogeriatric models. While contemporary guidelines increasingly acknowledge frailty and multimorbidity, they provide limited operational guidance regarding the competencies required to integrate these dimensions into cardiovascular expertise or who should assume responsibility for this integration in daily practice [3,7].

Against this background, the present narrative review addresses a foundational but underexplored question: who should be the cardiogeriatrician? Rather than advocating for a single professional profile, this work adopts a competency-based perspective, examining which clinical skills, interpretative capacities, and decision-making abilities are essential for delivering effective cardiogeriatric care in an ageing cardiovascular population. By analysing existing care models and the competencies they mobilise, we aim to provide a structured framework to inform clinical practice, training strategies, and health system organisation without prescribing a rigid or dogmatic solution.

This competency-based framework is intentionally conceptual and hypothesis-generating. It aims to describe what expertise is needed across clinical scenarios, rather than to claim superiority over existing cardiology- or geriatrics-led pathways.

Building on recent work defining the core competencies of geriatric cardiology, this review explores how these competencies can be operationalised across different professional and organisational models of care [11].

## 2. Methods

### 2.1. Study Design

This manuscript is a narrative review adopting a conceptual and competency-based approach. Its objective is not to perform a systematic or quantitative synthesis of evidence, but to integrate and interpret data from cardiology, geriatrics, and health services research in order to address a clinically relevant and unresolved question: how cardiogeriatric expertise should be defined and operationalised in an ageing cardiovascular population.

This approach was chosen to allow comparison of existing models of care, training pathways, and clinical practices across disciplines, and to derive a pragmatic framework centred on competencies rather than professional labels.

### 2.2. Literature Search Strategy

A structured literature search was performed in PubMed/MEDLINE on January 2026 to identify publications relevant to cardiogeriatric care and competency-based clinical practice in ageing cardiovascular populations. The search combined Medical Subject Headings (MeSH) and free-text keywords related to ageing, cardiovascular disease, geriatrics, frailty, multimorbidity, geriatric assessment, clinical competencies, decision-making, models of care, health services, and training. Publications were restricted to English language and to the period January 2000 to January 2026.

Search terms were combined using Boolean operators. Examples of search strings included: (i) (“cardiogeriatrics” OR cardiogeriatr* OR “geriatric cardiology”) AND (frailty OR “comprehensive geriatric assessment” OR multimorbid* OR “functional status”); (ii) (“heart failure” OR “atrial fibrillation” OR “structural heart disease”) AND (older OR elderly OR “very old”) AND (frailty OR multimorbid* OR cognition OR disability); and (iii) (competenc* OR “competency-based” OR “clinical competence”) AND (cardiology OR geriatrics) AND (older OR ageing) AND (“models of care” OR “health services” OR training).

To broaden coverage beyond database indexing, reference lists of key articles were manually screened, and major international guidelines, consensus documents, and scientific statements from cardiology and geriatrics societies were reviewed. Health services research and position papers describing integrated or interdisciplinary models of care were also considered. Because this is a narrative, conceptual review, we did not prospectively record the number of records retrieved at each step or reasons for exclusion, as no formal screening flow was planned. We did not perform duplicate screening nor a PRISMA flow diagram.

### 2.3. Study Selection and Scope

Publications were selected based on their relevance to at least one of the following domains: (i) cardiovascular disease management in older or frail adults; (ii) geriatric assessment, frailty, and functional reserve in cardiovascular care; (iii) models of collaboration or integration between cardiology and geriatrics; and (iv) competency-based approaches to clinical practice, training, or health system organisation.

Given the conceptual and interpretative nature of this review, no formal exclusion criteria were applied with respect to study design. Randomised controlled trials, observational studies, narrative reviews, consensus statements, and health services research were included when they provided clinically meaningful insights into cardiogeriatric practice.

To avoid over-representation of any single discipline, we purposively retained a balanced set of sources across cardiology, geriatrics, and health-services research, prioritising: (i) major international guidelines and consensus statements; (ii) foundational or frequently cited conceptual frameworks; and (iii) representative empirical studies describing cardiogeriatric or interdisciplinary care models. This purposive selection was intended to ensure conceptual coverage and applicability, rather than exhaustiveness. As a narrative review, this work is susceptible to selection bias; we aimed to mitigate this by using a structured search, cross-checking reference lists, and combining high-level guidance documents with representative empirical reports across multiple care models and competency domains.

### 2.4. Conceptual Framework and Synthesis

The selected literature was analysed qualitatively using a competency-based analytical framework. Rather than focusing on clinical outcomes or effect sizes, the synthesis aimed to:

Identify recurring clinical challenges in the care of very old cardiovascular patients;

Compare the strengths and limitations of existing cardiology- and geriatrics-led care models;

Derive a set of core clinical and decision-making competencies defining cardiogeriatric expertise.

This interpretative synthesis was used to move beyond specialty-based definitions and to propose a clinically grounded, transferable framework applicable across healthcare systems.

### 2.5. Ethical Considerations

This study is based exclusively on previously published data and does not involve human participants, identifiable patient information, or interventional procedures. Ethical approval and informed consent were therefore not required.

## 3. Why Cardiogeriatrics Is Not Just a Subspecialty

The development of cardiogeriatrics cannot be understood as the emergence of an additional cardiovascular subspecialty defined by disease entities or procedures. Rather, it reflects a growing mismatch between the organisation of cardiovascular care and the clinical reality of older frail patients, in whom outcomes are shaped as much by frailty, functional reserve, and vulnerability to harm as by cardiovascular pathophysiology itself [1,2,3,4].

Traditional cardiovascular subspecialisation is predominantly disease- and intervention-centred, with expertise structured around organs, technologies, or therapeutic classes. While this model has delivered major advances in selected populations, it shows clear limitations in very old adults, where identical cardiovascular interventions may yield markedly different benefits depending on functional status, cognitive reserve, multimorbidity, and treatment burden [2,4,6]. In this setting, technical success does not necessarily translate into meaningful clinical benefit.

Cardiogeriatrics therefore represents a shift in clinical reasoning rather than a narrow field of expertise. Its core contribution lies in integrating cardiovascular decision-making with geriatric principles—namely, the ability to balance expected benefit against functional risk, to interpret treatment response beyond biological or procedural endpoints, and to contextualise cardiovascular strategies within an individual’s overall clinical trajectory [6,7,8,9]. These dimensions are only partially addressed in contemporary guidelines, which increasingly acknowledge frailty but offer limited operational guidance for its integration into cardiovascular expertise [3,6].

Importantly, cardiogeriatrics does not compete with cardiology or geriatrics as established disciplines. Instead, it occupies the interface between them, addressing clinical questions that neither field can fully resolve in isolation. Cardiologists may lack formal training in geriatric assessment and functional evaluation, while geriatricians may not be sufficiently exposed to contemporary cardiovascular therapeutics, devices, and procedural decision-making [7,8,9,12].

Framing cardiogeriatrics as a subspecialty risks reinforcing siloed approaches and narrowing its scope to selected settings or expert centres. Conversely, a competency-based perspective acknowledges that cardiogeriatric expertise may be embodied by different professional profiles, provided that essential interpretative and decision-making skills are present. Recent work proposing core competencies of the modern geriatric cardiologist supports this view, shifting the focus from professional background to clinical capabilities aligned with the needs of an ageing cardiovascular population [10,13,14,15].

This distinction is critical to understanding why the central issue is not whether cardiogeriatrics should exist, but who should deliver it, on what basis, and with which competencies.

**Figure 1:**
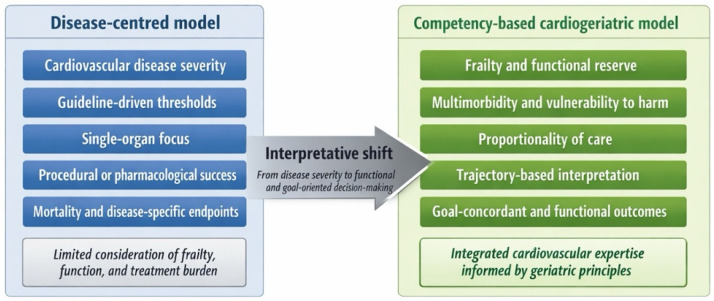
From disease-centred to competency-based cardiovascular care in older adults. Legends: Traditional cardiovascular care is primarily organised around disease severity, guideline-driven thresholds, and organ-centred endpoints. In very old adults, this approach may insufficiently capture the impact of frailty, functional reserve, multimorbidity, and vulnerability to harm. A competency-based cardiogeriatric model shifts clinical reasoning toward proportionality of care, trajectory-based interpretation, and goal-concordant decision-making, integrating cardiovascular expertise with geriatric principles to optimise meaningful functional outcomes beyond disease-specific endpoints.

This transition from disease-centred cardiovascular reasoning toward a competency-based cardiogeriatric approach is summarised in Figure 1.

## 4. Existing Models of Cardiogeriatric Care

Across healthcare systems, cardiogeriatric care has developed heterogeneously, largely driven by local expertise, institutional culture, and workforce availability rather than by standardised definitions or training pathways. As a result, several coexisting models can be identified, each mobilising different competencies and presenting distinct strengths and limitations [7,8,12]. In the sections below, we describe these models while explicitly noting the level of supporting evidence and key limitations, as outcome validation remains uneven across settings.

### 4.1. Cardiologist-Led Models with Geriatric Input

In many centres, cardiogeriatric care is delivered primarily by cardiologists who integrate selected geriatric principles into cardiovascular decision-making, often with ad hoc geriatric consultation. This model is frequently observed in heart failure (HF) clinics, structural heart programmes, and cardiovascular intensive care units, where cardiologists retain primary responsibility for diagnosis, treatment selection, and procedural decisions [7,8].

Its main strength lies in strong cardiovascular expertise and direct access to advanced diagnostics and interventions. However, geriatric assessment is often partial, inconsistently applied, or limited to advanced stages of care. As a result, functional vulnerability, cognitive impairment, or treatment burden may be under-recognised, particularly when clinical decisions are time-sensitive or procedure-driven [4,8,15]. This model relies heavily on individual clinician experience rather than on systematically acquired geriatric competencies.

Evidence supporting cardiologist-led models with geriatric input is primarily derived from observational reports and position statements, with limited comparative evaluation of clinical outcomes. Reported advantages often relate to feasibility and access to advanced cardiovascular care, whereas the consistency of geriatric assessment and its impact on patient-centred outcomes may vary across settings.

### 4.2. Geriatric-Led Models with Cardiovascular Expertise

An alternative approach places geriatricians at the centre of care for older adults with cardiovascular disease, particularly in post-acute settings, rehabilitation units, and long-term care facilities. In this model, cardiovascular management is integrated into comprehensive geriatric assessment, with an emphasis on function, comorbidity, and social context [5,9].

While this approach ensures robust evaluation of frailty, cognition, and functional outcomes, it may be limited by restricted access to specialised cardiovascular investigations or advanced therapies. Variability in cardiology training among geriatricians can also affect confidence in managing complex HF therapies, rhythm disorders, or structural heart disease, potentially leading to conservative or delayed interventions [13,16].

Published evidence on geriatric-led cardiovascular care is heterogeneous and largely observational, often reflecting local practice patterns and variable cardiology integration. While these models are well aligned with comprehensive geriatric assessment and functional outcomes, comparative data on hard cardiovascular endpoints and timely access to advanced therapies remain limited.

### 4.3. Multidisciplinary and Integrated Team Models

Multidisciplinary cardiogeriatric teams represent a more integrated model, combining cardiologists, geriatricians, nurses, and allied health professionals within shared care pathways. These programmes have been described in HF management, peri-procedural evaluation, and transitional care, aiming to balance cardiovascular benefit with functional preservation [6,9,17].

Such models facilitate shared expertise and improve recognition of geriatric syndromes, yet they remain resource-intensive and highly dependent on institutional support. Their scalability across diverse healthcare settings is uncertain, and their effectiveness may vary according to team composition, communication, and role clarity [12,17].

The evidence supporting multidisciplinary cardiogeriatric teams remains largely observational and context-dependent, with substantial heterogeneity in team composition, referral criteria, and delivered interventions. Reported benefits frequently rely on process indicators (e.g., coordination, discharge planning, access to assessment) rather than standardised comparative evidence on hard clinical endpoints. These limitations constrain generalisability and highlight the need for pragmatic comparative evaluations and implementation research.

### 4.4. Limitations of Model-Based Approaches

Although these models differ in structure and leadership, they share a common limitation: they define cardiogeriatric care primarily by who delivers it rather than by which competencies are required. This focus on professional background risks obscuring the essential clinical skills needed to manage very old patients with cardiovascular disease effectively. In addition, the current literature provides limited comparative outcome validation across these organisational models, and reported effects are difficult to interpret due to selection bias, implementation heterogeneity, and variable outcome definitions.

Moreover, model-based approaches are difficult to generalise across healthcare systems with differing workforce structures and training frameworks. In contrast, a competency-based perspective offers greater flexibility, allowing cardiogeriatric expertise to be developed and recognised across diverse professional profiles and care settings [11,18,19,20].

The following competency framework should be read as a descriptive lens to support clinical reasoning and collaboration, and not as a prescriptive care pathway or an outcome-validated model.

These observations support the need to move beyond model descriptions toward a structured analysis of the competencies that underpin effective cardiogeriatric care—a transition that forms the basis of the following section.

**Figure 2:**
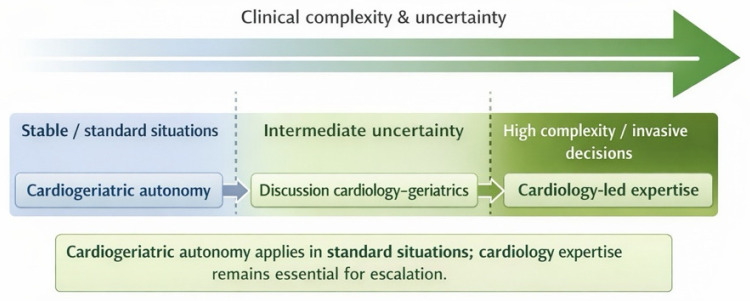
**Decision-making continuum according to clinical stability and complexity.** Legend: This figure illustrates a continuous decision-making spectrum in the care of older adults with cardiovascular disease, organised according to increasing clinical complexity and uncertainty. In stable or standard situations, cardiogeriatric autonomy allows independent management based on defined competencies. Situations of intermediate uncertainty require shared discussion between cardiology and geriatrics. In highly complex cases or when invasive decisions are considered, cardiology-led expertise becomes essential. This continuum highlights a non-normative, escalation-based approach in which cardiogeriatric autonomy applies in standard situations, while cardiology expertise remains crucial as complexity increases.

The articulation between cardiogeriatric autonomy and cardiology-led expertise across increasing clinical complexity is illustrated in Figure 2.

## 5. A Competency-Based Perspective on Cardiogeriatric Practice

The heterogeneity of existing cardiogeriatric models underscores the limitations of defining cardiogeriatric care by professional background or organisational structure alone. A competency-based perspective offers an alternative framework, shifting the focus from who delivers care to which clinical competencies are required to address the needs of older adults with cardiovascular disease [11,16,17].

Competency-based frameworks are increasingly used in medical education and health services research to describe complex clinical roles that transcend traditional disciplinary boundaries. Applied to cardiogeriatrics, this approach recognises that effective care depends on a specific set of interpretative, decisional, and communicative skills that may be acquired through different training pathways, provided that these competencies are explicitly identified and cultivated [21,22].

### 5.1. Core Interpretative Competencies

At the heart of cardiogeriatric practice lies the ability to interpret cardiovascular disease within the broader context of ageing. This is consistent with recent ESH consensus on hypertensive heart disease in older patients, which emphasises periodic frailty and functional assessment to guide treatment decisions [10]. This involves integrating frailty, functional status, cognitive impairment, and comorbidity into cardiovascular risk assessment and treatment selection [4,6,9]. Unlike standard cardiovascular practice, in which disease severity and prognostic scores often dominate decision-making, cardiogeriatric interpretation requires balancing expected cardiovascular benefit against vulnerability to harm and the risk of functional decline.

A further defining competency is the ability to recognise when conventional cardiovascular endpoints—such as symptom relief, biomarker improvement, or procedural success—do not translate into meaningful clinical benefit for the individual patient. Identifying discordance between biological response and functional outcome is therefore central to cardiogeriatric expertise, particularly in advanced-age patients with limited physiological reserve [5,6,11].

In practical terms, one workable profile for a cardiogeriatrician is a geriatrician with sustained, high-volume exposure to cardiovascular patients, who manages cardiovascular conditions on a daily basis and has developed independent clinical decision-making capacity for standard situations within clearly defined cardiogeriatric competencies.

Here, “standard situations” refers to clinically stable presentations managed within established HF and cardiovascular care frameworks, without diagnostic uncertainty, advanced structural disease, or indication for invasive evaluation.

For clarity, examples of “standard situations” include: (i) stable chronic HFrEF optimisation without haemodynamic instability or suspected advanced structural disease; (ii) routine diuretic adjustment for chronic congestion with a clear prior phenotype and monitoring plan; and (iii) anticoagulation review in atrial fibrillation incorporating falls risk and functional status when no complex electrophysiological or structural decision is pending. By contrast, cardiologist input is typically required in the presence of diagnostic uncertainty, suspected severe/complex valvular disease, refractory symptoms despite guideline-directed therapy, advanced device/interventional decisions, or acute instability.

Competence in core cardiovascular diagnostics—most notably focused, clinically oriented bedside echocardiography—represents a key enabling skill, as it reduces diagnostic delays, improves therapeutic responsiveness, and supports clinically grounded decisions in frail older adults. This focused echocardiographic competence is intended to support clinically oriented bedside decision-making and does not replace comprehensive cardiology-led imaging, particularly in complex valvular, diastolic, or structural heart disease.

In this context, focused bedside echocardiography refers to a limited decision-support protocol rather than comprehensive echocardiography. It typically targets gross LV systolic function, pericardial effusion, major congestion signs (e.g., IVC assessment), and obvious severe valvular pathology when clinically suspected, with a low threshold for referral to comprehensive cardiology-led imaging in case of uncertainty or complex structural disease. Training should follow local credentialing rules and include supervised practice with competency sign-off.

When these competencies are met, cardiogeriatric management allows independent decision-making in standard, protocol-based therapeutic situations, without routine cardiology consultation. Cardiology expertise remains essential and readily accessible for diagnostic confirmation in uncertain or complex cases, for treatment escalation, and for interventional or highly specialised care when indicated. Importantly, this framework does not seek to redefine professional scopes of practice, which remain governed by national regulations and institutional policies, but rather to clarify how cardiogeriatric competencies can be mobilised within existing collaborative care structures.

### 5.2. Decision-Making and Proportionality of Care

A second core competency concerns proportionality of care. In older frail patients, cardiovascular decisions frequently involve trade-offs between potential benefit, treatment burden, and risk of functional decline. Cardiogeriatric decision-making thus requires the capacity to tailor treatment intensity to functional reserve, recovery potential, and patient priorities rather than to disease stage alone [7,12,17].

This competency is particularly relevant in recurrent HF decompensations, invasive procedures, and escalation of disease-modifying therapies. Rather than applying uniform thresholds for intervention, cardiogeriatric practice emphasises context-sensitive decisions that may justify escalation, maintenance, or de-escalation of care based on expected functional yield [3,6,19].

In advanced illness, proportionality reasoning also includes timely consideration of therapy de-escalation and advance care planning when expected benefit becomes limited or burdensome. In such contexts, integrating palliative principles for refractory symptoms helps align cardiovascular strategies with comfort, function, and patient priorities.

### 5.3. Functional and Trajectory-Based Assessment

Cardiogeriatric competencies extend beyond single clinical encounters. Understanding a patient’s trajectory—whether stable, fluctuating, or declining—is essential to contextualise cardiovascular events and treatment responses. Functional recovery, tolerance to therapy, and resilience following physiological stress provide information that is often more informative than static baseline assessments in advanced age [5,18,23].

This longitudinal perspective does not require exclusive reliance on complex tools but depends on the clinician’s ability to synthesise repeated functional signals over time and to recognise transitions toward vulnerability or limited reversibility. Such interpretative capacity is central to anticipating outcomes and adjusting care strategies proactively [23,24].

### 5.4. Communication and Shared Decision-Making

Effective cardiogeriatric care also relies on advanced communication skills. Older patients with cardiovascular disease often prioritise maintaining independence, avoiding institutionalisation, or minimising treatment burden over marginal survival gains [25,26]. Translating complex cardiovascular information into meaningful, patient-centred discussions is therefore a core competency.

Shared decision-making in cardiogeriatrics requires framing options in terms of functional consequences and trade-offs rather than purely technical success. Clinicians must be able to support informed choices that align treatment strategies with patient values, particularly when prognosis is uncertain or benefit is incremental [25,26,27].

In older frail patients, goal-concordant care frequently requires interprofessional input beyond medicine alone. Physiotherapists, dietitians, occupational therapists, pharmacists, and social workers contribute to functional goal-setting, nutritional optimisation, medication reconciliation, discharge planning, and feasibility of treatment plans, thereby shaping what constitutes meaningful benefit and acceptable treatment burden.

### 5.5. Implications of a Competency-Based Framework

Adopting a competency-based perspective reframes the question of who should be the cardiogeriatrician. Rather than privileging a specific professional background, this framework recognises cardiogeriatric expertise as a set of competencies that can be developed by cardiologists, geriatricians, or hybrid practitioners within multidisciplinary teams.

This framework offers greater flexibility for training, workforce planning, and scalability across healthcare systems. It also provides a structured basis for evaluating cardiogeriatric practice, facilitating clearer expectations for clinicians and institutions engaged in the care of ageing cardiovascular populations [11,21,22,27].

To facilitate operationalisation, Table 1 provides illustrative examples of how key cardiogeriatric competencies may manifest in practice and how they may be assessed using established educational and quality-improvement approaches.

These examples are provided for illustration and are not intended as a standardised assessment framework.

**Figure 3:**
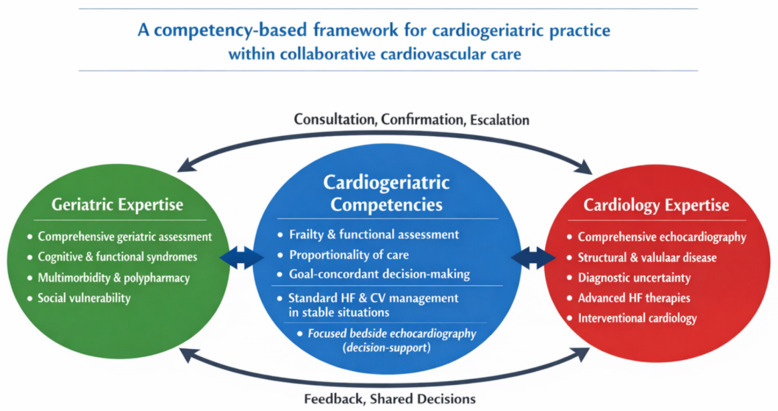
**A competency-based framework for cardiogeriatric practice within collaborative cardiovascular care.** Legend: Conceptual representation of a competency-based framework for cardiogeriatric practice within collaborative cardiovascular care. Core cardiogeriatric competencies include frailty and functional assessment, proportionality of care, goal-concordant decision-making, and standard cardiovascular management in clinically stable situations. Focused bedside echocardiography is shown as a decision-support tool and does not replace comprehensive cardiology-led imaging. The framework highlights complementary geriatric and cardiology expertise, with bidirectional interactions based on consultation, confirmation, escalation, and shared decision-making. The figure is conceptual and intended for hypothesis generation and evaluation, not as a validated decision tool.

Figure 3 illustrates a continuous decision-making spectrum rather than a prescriptive algorithm.

## 6. Implications for Training, Workforce, and Health Systems

A competency-based conceptualisation of cardiogeriatric practice has implications that extend beyond individual clinicians to education, workforce organisation, and health system design. As the cardiovascular population ages, the challenge is no longer limited to recognising geriatric vulnerability, but to ensuring that healthcare systems are equipped to integrate this dimension into cardiovascular decision-making at scale.

### 6.1. Rethinking Training Beyond Disciplinary Boundaries

Traditional training pathways in cardiology and geriatrics remain largely siloed, with limited structured exposure to the complementary discipline. As a result, cardiogeriatric competencies are often acquired informally and unevenly, depending on individual experience rather than explicit educational objectives. A competency-based framework offers a means to define shared learning outcomes—such as functional assessment, proportionality of care, and interpretation of treatment tolerance—that are relevant across disciplines [21,22].

Rather than advocating for the creation of a new specialty, this approach supports flexible educational strategies, including cross-disciplinary rotations, focused curricula, and continuing professional development aligned with clearly articulated competencies. Such modular approaches may be more adaptable and scalable than highly specialised fellowship models, particularly in healthcare systems facing workforce constraints [21,22].

From an implementation perspective, cardiogeriatric competencies are not expected to arise spontaneously but may be acquired through structured yet flexible clinical exposure rather than through rigid specialty pathways. Practical avenues could include targeted cross-disciplinary rotations (e.g., geriatrics within HF or structural cardiology programmes), focused training in core diagnostic skills such as bedside echocardiography, and supervised participation in multidisciplinary cardiovascular decision-making for frail older adults. Importantly, these competencies are likely to develop through sustained clinical volume and longitudinal responsibility for cardiovascular patients, rather than through isolated educational interventions or formal certification alone.

### 6.2. Workforce Organisation and Role Clarity

From a workforce perspective, cardiogeriatric care is unlikely to be delivered by a single professional profile in most settings. Instead, it will rely on diverse configurations of cardiologists, geriatricians, nurses, and allied health professionals who collectively mobilise cardiogeriatric competencies. A competency-based framework helps shift the focus from professional titles to functional roles, clarifying responsibilities within interdisciplinary teams and supporting more consistent care delivery [17,19].

This perspective also acknowledges that cardiogeriatric expertise may be distributed across care settings rather than concentrated in specialised centres. Such flexibility is essential to ensure equitable access to cardiogeriatric-informed cardiovascular care, particularly in regions with limited specialist availability or high demographic ageing pressure [22,23].

Nurses and allied health professionals often operationalise cardiogeriatric care through functional status screening, patient education, therapy monitoring, medication reconciliation, and early identification of adverse effects or decompensation. Defining shared competencies (e.g., structured functional prompts, self-care coaching, monitoring protocols, escalation criteria) may improve implementation fidelity and continuity across settings.

### 6.3. Health System Design and Scalability

Health systems face increasing pressure to deliver complex cardiovascular care to older adults within constrained resources. Models that depend on highly specialised professionals or centralised expertise may prove difficult to scale. In contrast, a competency-based approach aligns with broader trends toward integrated, patient-centred care and task-sharing across disciplines [21,24].

By embedding cardiogeriatric competencies across multiple points of care—outpatient clinics, HF programmes, peri-procedural pathways, and post-acute services—health systems may enhance continuity and coherence of care without relying exclusively on episodic specialist input. This approach prioritises clinical reasoning capacity over structural complexity.

### 6.4. Implications for Quality Metrics and Policy

Finally, a competency-based framework has implications for how quality of care is defined and assessed. Conventional cardiovascular quality indicators emphasise disease-specific outcomes and procedural metrics, which may inadequately capture what constitutes meaningful benefit in very old patients. Complementing these indicators with measures reflecting functional outcomes, treatment burden, and goal concordance would better align evaluation frameworks with cardiogeriatric principles [24,25,27].

Such an evolution does not require abandoning guideline-based cardiovascular care, but rather contextualising it within outcome frameworks that reflect the priorities and vulnerabilities of ageing populations. Explicit recognition of cardiogeriatric competencies may also inform credentialing, service evaluation, and policy discussions around workforce planning in ageing societies.

### 6.5. Digital Health and Telemedicine

Remote monitoring and telemedicine may support scalability of cardiogeriatric competencies by enabling earlier detection of decompensation, structured follow-up, and multidisciplinary review across sites. Digital tools can also support competency development through case libraries, supervised tele-consultations, and standardised documentation of functional and goal-oriented outcomes. Such approaches may improve access to cardiogeriatric-informed care in territories with limited specialist density, provided interoperability and governance constraints are addressed.

## 7. Discussion

This narrative review addresses a foundational yet insufficiently formalised question in contemporary cardiovascular care: who should deliver cardiogeriatric expertise in an ageing cardiovascular population? Rather than proposing a new subspecialty or privileging a specific professional background, our analysis supports a competency-based interpretation of cardiogeriatric practice, grounded in the clinical realities of older frail patients with cardiovascular disease.

### 7.1. Moving Beyond Professional Identity

Existing cardiogeriatric models demonstrate that effective care for older adults with cardiovascular disease can emerge from diverse organisational structures, including cardiology-led, geriatrics-led, and multidisciplinary approaches [7,8,18]. However, defining cardiogeriatrics primarily through professional identity risks obscuring the central issue: the ability to integrate cardiovascular decision-making with geriatric principles such as functional assessment, proportionality of care, and vulnerability to harm [4,6,9].

A competency-based perspective shifts the focus from who delivers care to how clinical decisions are interpreted and contextualised. This approach aligns with broader trends in complex care, where clinical capability increasingly transcends disciplinary boundaries and is defined by interpretative and decisional skills rather than by specialty labels alone [21,22].

This competency-based framework does not imply a redistribution of professional roles, but rather clarifies how cardiology and geriatrics can interact more effectively: cardiogeriatric autonomy applies to standard decision-making in frail older adults, while cardiology expertise remains essential for diagnostic confirmation, complex cases, and interventional care.

Importantly, this framework is not intended to claim clinical superiority over cardiology-led or geriatrics-led models; it provides a competency-based description of capabilities that may be distributed across professionals and teams.

### 7.2. Addressing the Gap Between Guidelines and Clinical Practice

Cardiovascular guidelines increasingly acknowledge frailty, multimorbidity, and geriatric syndromes as modifiers of prognosis and treatment response [3,6]. Nevertheless, they provide limited operational guidance on how these dimensions should be integrated into cardiovascular expertise or who should assume responsibility for this integration in routine practice. This gap contributes to heterogeneity in care delivery and uncertainty in decision-making for advanced-age patients, particularly in situations where evidence is sparse or competing risks dominate.

The framework proposed in this review does not challenge guideline-based cardiovascular care but rather complements it by offering a structured interpretative layer. By articulating the competencies required to operationalise geriatric principles within cardiovascular practice, this approach may help translate high-level recommendations into clinically meaningful reasoning without imposing rigid pathways [11,17].

### 7.3. Implications for Clinical Decision-Making in Advanced Age

A central implication of a competency-based cardiogeriatric framework lies in its potential to improve clinical decision-making in situations characterised by uncertainty and trade-offs. In older adults with frailty, cardiovascular interventions that are technically successful may nonetheless result in functional decline, loss of autonomy, or disproportionate treatment burden [5,6,23,24]. Interpreting treatment response through a functional and goal-oriented lens is therefore essential to distinguish meaningful benefit from biological improvement alone [25,27].

By emphasising competencies related to proportionality, functional yield, and communication, cardiogeriatric practice aligns cardiovascular strategies with patient priorities, particularly when survival gains are marginal or uncertain. This perspective is consistent with patient goal-directed care models and shared decision-making frameworks increasingly advocated in geriatric medicine [25,26,27].

Decision-making in advanced age often involves ethical tensions between potential benefit, treatment burden, and risk of functional harm. A competency-based cardiogeriatric approach supports explicit proportionality reasoning under uncertainty, timely ACP, and appropriate de-escalation when burdens outweigh benefits. This includes the ability to integrate palliative care principles for refractory symptoms and to align cardiovascular strategies with comfort, function, and patient priorities when prognosis is limited or when expected benefit is incremental.

### 7.4. Training, Workforce, and System-Level Considerations

From a system perspective, defining cardiogeriatrics by competencies rather than by professional titles offers greater flexibility for training and workforce organisation. Given demographic ageing and specialist shortages, it is unlikely that cardiogeriatric care can be delivered exclusively by highly specialised clinicians or centralised centres [21,22]. A competency-based framework allows cardiogeriatric expertise to be distributed across care settings and professional profiles, improving scalability and equity of access.

This approach is also compatible with contemporary educational models that emphasise competency acquisition, cross-disciplinary learning, and continuing professional development rather than rigid specialty boundaries [21,22,28]. Explicit recognition of cardiogeriatric competencies may therefore support curriculum development, credentialing, and quality improvement initiatives adapted to ageing populations.

Real-world adoption of competency-based cardiogeriatric care may be limited by regulatory and scope-of-practice constraints that differ across countries and institutions, including accountability for prescribing, imaging, and escalation decisions. Financial and reimbursement models may also misalign incentives, as multidisciplinary assessment, longitudinal coordination, and goal-oriented discussions are time-intensive yet variably recognised in payment structures.

Workforce limitations remain a major barrier, including availability of geriatricians, access to comprehensive geriatric assessment resources, and protected time for cross-disciplinary training; advanced practice nurses and allied health professionals may help, but their roles and authorisations are highly system-dependent. Implementation may also be uneven across territories, with greater feasibility in tertiary centres than in peripheral or rural settings where specialist density, diagnostics, and multidisciplinary infrastructure are limited. These constraints support adaptable, context-specific implementation strategies and reinforce the need to evaluate not only clinical outcomes, but also feasibility, staffing requirements, and sustainability across health systems.

Implementing competency-based cardiogeriatric care may increase upfront resource use (assessment time, coordination, training), but may plausibly reduce downstream costs related to adverse drug events, misaligned interventions, and unplanned hospitalisations. Formal economic evaluation is needed and should be embedded in pragmatic implementation studies alongside patient-centred outcomes.

Although examples may differ across healthcare systems, the competency-based framework proposed here is intended to be transferable across organisational contexts; however, implementation is inherently context-dependent and may require competencies to be distributed across multidisciplinary teams and tailored to local regulations, workforce availability, and reimbursement structures.

### 7.5. Limitations and Future Directions

This framework is conceptual and hypothesis-generating and does not claim outcome superiority over existing cardiology- or geriatrics-led pathways in the absence of comparative studies. As a narrative synthesis, it is also susceptible to selection bias; we sought to mitigate this through a structured search approach, manual screening of reference lists, and purposive inclusion of complementary source types (guidelines and consensus documents, conceptual frameworks, and representative empirical reports).

Empirical evaluation is required before recommending widespread implementation. Pragmatic assessments should compare competency-based implementation strategies with usual care across diverse settings and include a balanced outcome set: hard clinical endpoints (e.g., hospitalisations, adverse drug events, procedure-related complications), geriatric and patient-centred outcomes (e.g., functional trajectory, quality of life, treatment burden, goal concordance), and implementation measures (e.g., feasibility, fidelity, acceptability, role clarity, workforce requirements) that condition scalability.

Potential evaluation approaches include: (i) pragmatic comparative studies of competency-based implementation versus usual care; (ii) stepped-wedge or cluster designs for service-level implementation across sites; and (iii) mixed-methods implementation studies assessing context, fidelity, and scalability alongside patient-centred outcomes.

Several additional limitations should be noted. The relative contribution of individual competencies to outcomes remains to be quantified, and implementation is likely to be context-dependent across healthcare systems, particularly where access to cardiovascular diagnostics or structured cardiology collaboration is limited. Future research should therefore clarify whether competency-based cardiogeriatric approaches improve decision quality and patient-reported outcomes in older cardiovascular populations, using clinically meaningful endpoints rather than exclusively disease-centred metrics [18,23].

Additional risks include diffusion of responsibility when competencies are distributed across teams without explicit role clarity and escalation pathways, and implementation friction in highly siloed systems with rigid specialty boundaries. Moreover, long-term outcome effects of competency-based implementation remain unknown and may depend strongly on context and fidelity.

Applicability may also be influenced by cultural and regional factors shaping attitudes to ageing, family involvement, and patient autonomy, which can affect how goals are elicited and how proportionality is judged. Implementation should therefore be sensitive to local norms and decision-making cultures.

## 8. Conclusions

As cardiovascular populations continue to age, delivering effective and proportionate care to advanced-age patients cannot rely on disease-centred models alone. Cardiovascular decision-making increasingly takes place at the intersection of multimorbidity, frailty, functional vulnerability, and competing priorities, revealing the limitations of rigid specialty boundaries when evidence is sparse and trade-offs are prominent.

Rather than proposing a new subspecialty or privileging a single professional profile, this review offers a competency-based framework to clarify what cardiogeriatric expertise entails and who may deliver it. By shifting attention from professional identity to clinical capability, the framework highlights core skills needed to integrate cardiovascular expertise with geriatric principles, including frailty- and function-informed interpretation, proportionality of care, trajectory-based reasoning, and goal-concordant decision-making.

This framework is intended to support clinical reasoning and collaboration, not to prescribe care pathways or replace guideline-based recommendations. By providing a structured lens to recognise, develop, and deploy cardiogeriatric competencies across diverse care settings, it aims to inform practice, training, and health system organisation as the needs of ageing cardiovascular populations evolve.

### Practical Implications

For clinicians, a competency-based lens can be implemented by routinely integrating frailty/functional assessment, explicit proportionality reasoning, and documentation of patient priorities when choosing therapies, with clear escalation rules for diagnostic uncertainty or invasive decisions. For educational institutions, training may be structured around modular competencies (frailty assessment, goal-concordant communication, trajectory-based review, medication optimisation) supported by supervised clinical exposure, case-based learning, and structured workplace-based assessment. For administrators, quality assessment should combine clinical outcomes with patient-centred and implementation metrics (functional trajectory, treatment burden, goal concordance, feasibility, fidelity, and staffing requirements), enabling context-specific scaling across services.

Key Messages: Population ageing is transforming cardiovascular care, with frailty, multimorbidity, and functional vulnerability becoming major determinants of outcomes in older adults with frailty.

Cardiogeriatrics should be defined less by professional background than by the competencies required to deliver proportionate, patient-centred cardiovascular care in older adults. Existing cardiogeriatric care models remain heterogeneous and are often organised around roles rather than explicit competency expectations.

A competency-based framework clarifies core requirements, including frailty and functional assessment, proportionality of care, trajectory-based interpretation, and goal-concordant decision-making. Cardiogeriatric expertise can be embodied by different professional profiles, including geriatricians with sustained cardiovascular practice and clearly defined competencies. The framework supports collaborative care: cardiogeriatric autonomy applies in standard, clinically stable situations, while cardiology expertise remains essential for diagnostic uncertainty, advanced disease, and interventional decision-making.

## Figures and Tables

**Table 1:** Key cardiogeriatric competencies, manifestations in practice, and illustrative assessment methods.

Competency	Manifestation in Practice	Assessment Methods (Examples)
Frailty- and function-informed interpretation	Integrates frailty/functional status into cardiovascular risk–benefit appraisal and treatment selection	Direct observation (mini-CEX), case-based discussion, chart audit
Proportionality of care	Tailors treatment intensity to physiological reserve, competing risks, and patient priorities	Multidisciplinary case review, peer assessment, clinical audit
Trajectory-based reasoning	Uses longitudinal functional signals to guide escalation, maintenance, or de-escalation	Portfolio/logbook, longitudinal supervision, morbidity and mortality review
Communication and shared decision-making	Frames options as trade-offs; documents goals and preferences	Observed encounters, structured feedback, patient experience measures
Medication optimisation in multimorbidity	Adjusts therapies considering polypharmacy, renal risk, falls risk, and tolerability	Prescribing audit, pharmacist review, simulation
Focused bedside echocardiography (decision-support)	Applies a limited bedside protocol to support standard decisions and triggers referral when uncertainty/complexity is present	Skills checklist, supervised scan logbook, competency sign-off
Serious illness communication and advance care planning	Elicits goals/values, documents advance care planning (ACP), anticipates de-escalation triggers, integrates palliative principles for refractory symptoms	Observed encounter, structured feedback, chart audit, multidisciplinary case review

## Data Availability

The raw data supporting the conclusions of this article will be made available by the authors on request.

## Abbreviations

The following abbreviations are used in this manuscript:

ACP	Advance Care Planning
ESC	European Society of Cardiology
ESH	European Society of Hypertension
HF	Heart Failure
HFrEF	Heart Failure with reduced Ejection Fraction
MEDLINE	Medical Literature Analysis and Retrieval System Online
Mini-CEX	Mini-Clinical Evaluation Exercise
MeSH	Medical Subject Headings

## References

[1] North B.J., Sinclair D.A. (2012). The intersection between aging and cardiovascular disease. Circ. Res..

[2] Metra M., Teerlink J.R. (2017). Heart failure. Lancet.

[3] McDonagh T.A., Metra M., Adamo M., Gardner R.S., Baumbach A., Böhm M., Burri H., Butler J., Čelutkienė J., Chioncel O. (2023). 2023 Focused Update of the 2021 ESC Guidelines for the diagnosis and treatment of acute and chronic heart failure. Eur Heart J..

[4] Afilalo J., Alexander K.P., Mack M.J., Maurer M.S., Green P., Allen L.A., Popma J.J., Ferrucci L., Forman D.E. (2014). Frailty assessment in the cardiovascular care of older adults. J. Am. Coll. Cardiol..

[5] Boyd C.M., Landefeld C.S., Counsell S.R., Palmer R.M., Fortinsky R.H., Kresevic D., Burant C., Covinsky K.E. (2008). Recovery of activities of daily living in older adults after hospitalization for acute medical illness. J. Am. Geriatr. Soc..

[6] Vitale C., Jankowska E., Hill L., Piepoli M., Doehner W., Anker S.D., Lainscak M., Jaarsma T., Ponikowski P., Rosano G.M.C. (2019). Heart Failure Association/European Society of Cardiology position paper on frailty in patients with heart failure. Eur. J. Heart Fail..

[7] Dodson J.A., Matlock D.D., Forman D.E. (2016). Geriatric Cardiology: An Emerging Discipline. Can. J. Cardiol..

[8] Damluji A.A., Forman D.E., van Diepen S., Alexander K.P., Page R.L., Hummel S.L., Menon V., Katz J.N., Albert N.M., Afilalo J. (2020). Older Adults in the Cardiac Intensive Care Unit: Factoring Geriatric Syndromes in the Management, Prognosis, and Process of Care: A Scientific Statement from the American Heart Association. Circulation.

[9] Alexander K.P., Newby L.K., Cannon C.P., Armstrong P.W., Gibler W.B., Rich M.W., Van de Werf F., White H.D., Weaver W.D., Naylor M.D. (2007). Acute coronary care in the elderly, part I: Non-ST-segment-elevation acute coronary syndromes: A scientific statement for healthcare professionals from the American Heart Association Council on Clinical Cardiology: In collaboration with the Society of Geriatric Cardiology. Circulation.

[10] Camafort M., Kasiakogias A., Agabiti-Rosei E., Masi S., Iliakis P., Benetos A., Jeong J.O., Lee H.Y., Muiesan M.L., Sudano I. (2025). Hypertensive heart disease in older patients: Considerations for clinical practice. Eur. J. Intern. Med..

[11] Esser R., Mondragon A., Larbaneix M., Esteban M., Farges C., Nisse Durgeat S., Maurou O., Harboun M. (2026). Core Competencies of the Modern Geriatric Cardiologist: A Framework for Comprehensive Cardiovascular Care in Older Adults. J. Clin. Med..

[12] Rich M.W., Chyun D.A., Skolnick A.H., Alexander K.P., Forman D.E., Kitzman D.W., Maurer M.S., McClurken J.B., Resnick B.M., Shen W.K. (2016). Knowledge gaps in cardiovascular care of the older adult population: A scientific statement from the American Heart Association, American College of Cardiology, and American Geriatrics Society. J. Am. Coll. Cardiol..

[13] Forman D.E., Alexander K., Brindis R.G., Curtis A.B., Maurer M., Rich M.W., Sperling L., Wenger N.K. (2016). Improved Cardiovascular Disease Outcomes in Older Adults. F1000Research.

[14] Rich M.W. (2002). Management of heart failure in the elderly. Heart Fail. Rev..

[15] Damluji A.A., Chung S.E., Xue Q.L., Hasan R.K., Moscucci M., Forman D.E., Bandeen-Roche K., Batchelor W., Walston J.D., Resar J.R. (2021). Frailty and cardiovascular outcomes in the National Health and Aging Trends Study. Eur. Heart J..

[16] Forman D.E., Rich M.W., Alexander K.P., Zieman S., Maurer M.S., Najjar S.S., Cleveland J.C., Krumholz H.M., Wenger N.K. (2011). Cardiac care for older adults. Time for a new paradigm. J. Am. Coll. Cardiol..

[17] Dunlay S.M., Chamberlain A.M. (2016). Multimorbidity in Older Patients with Cardiovascular Disease. Curr. Cardiovasc. Risk Rep..

[18] Damluji A.A., Forman D.E., Wang T.Y., Chikwe J., Kunadian V., Rich M.W., Young B.A., Page R.L., DeVon H.A., Alexander K.P. (2023). Management of Acute Coronary Syndrome in the Older Adult Population: A Scientific Statement from the American Heart Association. Circulation.

[19] Afilalo J., Mottillo S., Eisenberg M.J., Alexander K.P., Noiseux N., Perrault L.P., Morin J.F., Langlois Y., Ohayon S.M., Monette J. (2012). Addition of frailty and disability to cardiac surgery risk scores identifies elderly patients at high risk of mortality or major morbidity. Circ. Cardiovasc. Qual. Outcomes.

[20] Frank J.R., Snell L.S., Cate O.T., Holmboe E.S., Carraccio C., Swing S.R., Harris P., Glasgow N.J., Campbell C., Dath D. (2010). Competency-based medical education: Theory to practice. Med. Teach..

[21] Ten Cate O. (2017). Competency-Based Postgraduate Medical Education: Past, Present and Future. GMS J. Med. Educ..

[22] Gill T.M., Gahbauer E.A., Han L., Allore H.G. (2010). Trajectories of disability in the last year of life. N. Engl. J. Med..

[23] Boyd C.M., Xue Q.L., Simpson C.F., Guralnik J.M., Fried L.P. (2005). Frailty, hospitalization, and progression of disability in a cohort of disabled older women. Am. J. Med..

[24] Fried T.R., McGraw S., Agostini J.V., Tinetti M.E. (2008). Views of older persons with multiple morbidities on competing outcomes and clinical decision-making. J. Am. Geriatr. Soc..

[25] Bernacki R.E., Block S.D., American College of Physicians High Value Care Task Force (2014). Communication about serious illness care goals: A review and synthesis of best practices. JAMA Intern. Med..

[26] Tinetti M.E., Naik A.D., Dodson J.A. (2016). Moving from Disease-Centered to Patient Goals-Directed Care for Patients with Multiple Chronic Conditions: Patient Value-Based Care. JAMA Cardiol..

[27] Reuben D.B., Tinetti M.E. (2012). Goal-oriented patient care—An alternative health outcomes paradigm. N. Engl. J. Med..

[28] Ouslander J.G., Berenson R.A. (2011). Reducing unnecessary hospitalizations of nursing home residents. N. Engl. J. Med..

